# Postoperative D-dimer levels predict venous thromboembolisms detected with contrast-enhanced computerized tomography in patients undergoing anterior cruciate ligament reconstruction

**DOI:** 10.1186/s12891-023-06212-4

**Published:** 2023-02-06

**Authors:** Yusuke Hashimoto, Eriko Komiya, Kazuya Nishino, Yohei Nishida, Atsushi Masuda, Hiroaki Nakamura

**Affiliations:** 1 Department of Orthopaedic Surgery, Osaka Metropolitan University Graduate School of Medicine, 1-4-3 Asahi-machi, Abeno-ku, Osaka, 545-8585 Japan; 2 Department of Orthopaedic Surgery, Osaka City University Graduate School of Medicine, Osaka, Japan; 3 Department of Orthopaedic Surgery, Saiseikai Nakatsu Hospital, Osaka, Japan

**Keywords:** Venous thromboembolism, Anterior cruciate ligament reconstruction, Contrast-enhanced computerized tomography, Postoperative D-dimer level

## Abstract

**Background:**

In the literature, factors associated with postoperative venous thromboembolisms (VTEs) after anterior cruciate ligament reconstruction (ACLR) are limited. This study aimed to investigate the incidence of venous thromboembolisms (VTEs) after anterior cruciate ligament reconstruction (ACLR) and to identify risk and predictive factors for VTEs.

**Methods:**

This retrospective study included 136 patients who underwent arthroscopic ACLR with mechanical prophylaxis between April 2012 and July 2022. Contrast-enhanced computed tomography (CT) was applied to detect VTEs comprising deep venous thromboses and pulmonary embolisms 7 days after surgery. Data including age, sex, body mass index, concomitant treatments, graft types, smoking status, operative and tourniquet times, postoperative D-dimer levels, and other laboratory test results, were collected for analyses. The incidence of radiographically confirmed VTEs and the associated risk factors, such as age, sex, body mass index, concomitant treatments, graft types, smoking status, operative and tourniquet times, postoperative D-dimer levels, and other laboratory test results, were analyzed.

**Results:**

The overall incidence of radiographic VTEs was 11.0% (15 cases) in 136 patients. There was one symptomatic patient who had Homan’s sign. Multivariable analysis indicated that postoperative D-dimer level was an independent factor related to a radiographic VTE after ACLR, although there was no association between radiographic VTEs and preoperative status or operation status. The optimal cutoff value for postoperative D-dimer level was 2.8 μg/ml according to the receiver operating characteristic curve analysis, with a sensitivity of 80.0% and specificity of 83.5%.

**Conclusion:**

The incidence of ACLR-associated radiographical VTEs (deep venous thrombosis and pulmonary embolism) under mechanical prophylaxis was 11.0% in this study. An elevated D-dimer level at 7 days after surgery is an independent predictor of VTE in patients undergoing ACLR. The postoperative D-dimer level is a more reliable marker for identifying VTE in patients who underwent ACLR.

## Background

Anterior cruciate ligament reconstruction (ACLR) is a common orthopedic procedure, particularly in a relatively young and athletic population. Various postoperative complications have been reported to be associated with ACLR, one of which is venous thromboembolism (VTE), including deep venous thrombosis (DVT) and pulmonary embolism (PE) [1–5]. DVT may develop into a life-threatening PE. Currently, regarding the incidence of VTE being associated with ACLR, the incidence of VTE after ACLR varies from 0.4 to 16.4% [6–12]. However, there is little information on DVT and PE rates as well as protocols for VTE prophylaxis in patients undergoing ACL reconstruction. There is no standard of care, including antithrombotic medicine, for VTE after ACL reconstruction [13–15]. There are reports of cases with fatal or massive PE after ACLR [16–18]. Thus, the detection of PE after ACLR should be considered an urgent issue. Contrast-enhanced CT has improved the diagnosis of PE [19, 20] and DVT [21–24] by facilitating the determination of the incidence of DVT along with PE. and has a sensitivity and specificity similar to sonography in diagnosing proximal DVT of the lower extremities in patients with suspected PE [25]. Considering the morbidity and potential mortality associated with VTE events, a better understanding of its prevalence after ACL reconstruction is vital [7]. D-dimer level is widely used to diagnose thromboses, such as DVT and PE because it is a marker of endogenous fibrinolysis by plasmin [26–28]. However, it should be considered that D-dimer levels can be elevated under several circumstances, such as tumors, surgery, chemoradiotherapy, inflammation, and advanced age [29]. Evaluating the clinical risk factors, including D-dimer levels associated with the incidence of postoperative VTEs, can help with better management of patients with ACL reconstruction.

The aims of this study were to investigate the incidence of VTEs after ACLR using contrast-enhanced CT to determine potential risk factors and to determine whether postoperative D-dimer levels could be used to predict a VTE in patients undergoing ACLR. We hypothesized that the postoperative D-dimer level would be a more reliable marker for identifying patients who developed VTE after ACL reconstruction.

## Material and methods

This study was approved by the Ethical Committee of Osaka Metropolitan University Graduate School of Medicine, and written informed consent was obtained from all enrolled participants. All procedures performed in this study involving human participants were in accordance with the 1964 Helsinki Declaration and its later amendments or comparable ethical standards. Consecutive patients who underwent ACL reconstruction from April 2012 to July 2022 were retrospectively evaluated with the medical records, including CT findings. All patients routinely underwent postoperative CT scans for bone foramen evaluation. In addition, contrast-enhanced searching for VTE was performed for the patients who accepted this study. The inclusion criteria were age of > 20 years, having undergone ACL reconstruction and patients who were explained to examine the Contrast-enhanced CT 7 days after ACL reconstruction. The exclusion criteria were allergy to iodine; a history of asthma; renal insufficiency, that is, eGFR value of < 30; lack of agreement to participate in this study; patients who were given chemical DVT prophylaxis before surgery and patients without a postoperative D-dimer laboratory blood test. Contrast-enhanced CT was performed on postoperative day 7 after ACL reconstruction.

Details such as age, sex, body side, body mass index (BMI), smoking status, graft selection, concomitant treatments, duration of operation, length of tourniquet use, and blood test data (preoperative data included white blood cells, hemoglobin, platelets, prothrombin time-international normalized ratio (PT-INR), and D-dimer level, and D-dimer level on postoperative day 7) were recorded.

### Surgical procedure and postoperative rehabilitation

All surgeries were performed by a single surgeon under general anesthesia and the leg to be operated on was placed in a lithotomy position or supine position. A tourniquet was secured in the proximal femur, and it was inflated only when a graft was harvested or during meniscus suturing. We inflated the tourniquet as minimum required; it was deflated for procedures lasting for > 90 min and re-inflated 15 min later if necessary. The transportal double-bundle technique was performed with a hamstring autograft and transtibial single bundle technique with a quadriceps tendon autograft and bone-patellar tendon-bone autograft. A suction drain was placed at the tendon harvest site and removed within 24 h after the surgery. No postoperative antithrombotic medicine was used. All patients used a sequential compressive device on the foot on the operative day and compressive stockings were used from the day after the surgery to postoperative day 14. At 24 h postoperatively, the patients began a rehabilitation regimen that included isometric quadriceps muscle strengthening and a straight leg-raising exercise with a knee immobilization brace for 1 week postoperatively. Partial weight-bearing and knee range-of-motion exercises were allowed at 1 week postoperatively; full weight-bearing was allowed at 5 weeks; and participation in low-impact activities (including jogging) was allowed at 4 months. Vigorous sports activities were allowed at approximately 8 months postoperatively.

### CT examination

**Fig. 1:**
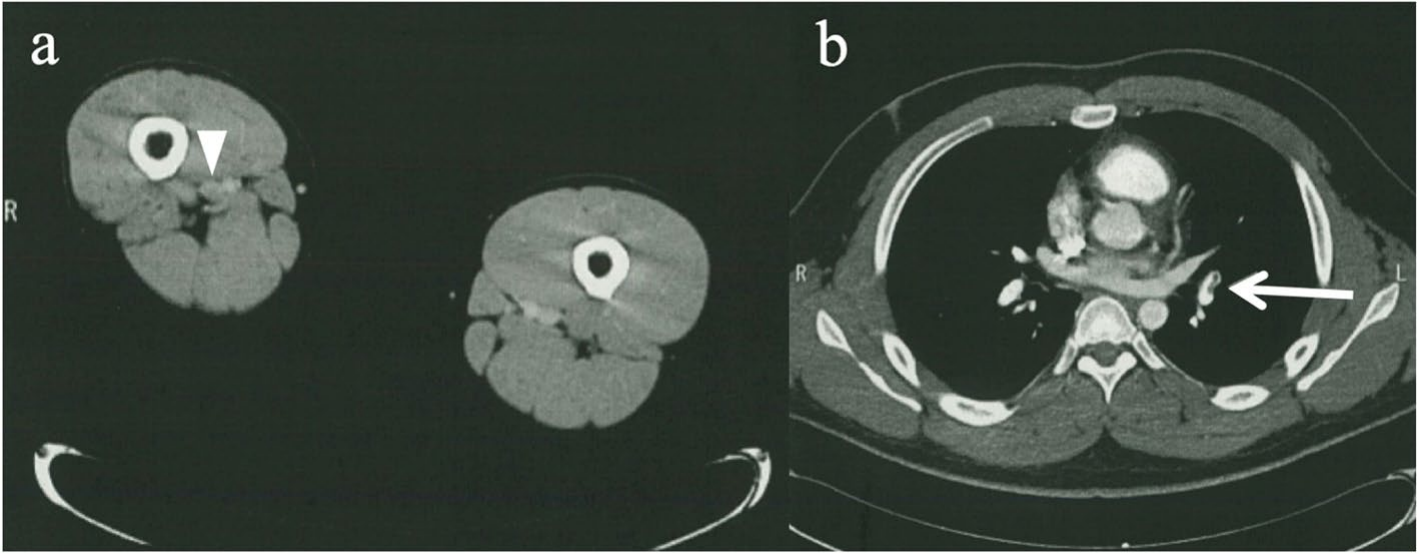
Contrast-enhanced CT was read by radiology specialists to obtain a definitive diagnosis of DVT (**a**; arrowhead) and PE (**b**; arrow)

Contrast-enhanced CT was performed 7 days after surgery using a 64-row multi-slice CT (VCT VISION®; General Electric Company, USA or SOMATOM sensation 64®; Siemens Healthcare, Germany). Contrast-enhanced CT images of the thoracic large vessel and lower extremity vein of all cases were obtained. The contrast agent (Omnipaque®, Daiichi Sankyo Company, Japan) was injected into an upper-extremity vein. During the arterial phase, for 25–40 s after injection, imaging was performed from the pulmonary apex to the costophrenic angle. During the venous phase, 240 s after injection of the contrast agent, imaging was performed from the diaphragm to the foot. All images were acquired as 3-mm horizontal axial sections, from which coronal and sagittal sections were reconstructed. The contrast-enhanced CT was read by radiology specialists to obtain a definitive diagnosis of DVT (Fig. 1a) and PE (Fig. 1b).

Once the presence of thrombus was confirmed, the patients were treated with anticoagulation therapy according to the instructions of the cardiologist. Patients who had VTE received an anticoagulant for 3 months after surgery and they were reexamined with contrast-enhanced CT to evaluate the VTE.

### Statistical analysis

A Student’s *t*-test was used for continuous variables (age, BMI, operation time, tourniquet time, and blood test). A χ^2^ test or Fisher’s exact test was used for categorical variables (sex, smoking status, operation technique, and graft). Multivariate logistic regression analysis was performed to adjust for the confounding factors with *P*-value < 0.1 in the univariate analysis and the risk factors associated with VTE in the previous study [7, 8, 30]. Significance was set at *P* = 0.05. Receiver operating characteristic (ROC) curves were also applied to determine the cutoff value with regard to the statistically significant factor(s). The area under the curve (AUC) was also calculated from ROC curves and the cutoff value was determined. A power analysis was performed with the power (α) difference and standard deviations set at 0.8, 0.05, 1.54, and 1.3, according to the D-dimer level. The analysis revealed that a minimum of 60 patients were required for a *t*-test to detect a difference between the VTE negative and VTE positive patients. All statistical analyses were performed with R version 7.0 (The R Foundation for Statistical Computing, Vienna, Austria).

## Results

**Fig. 2:**
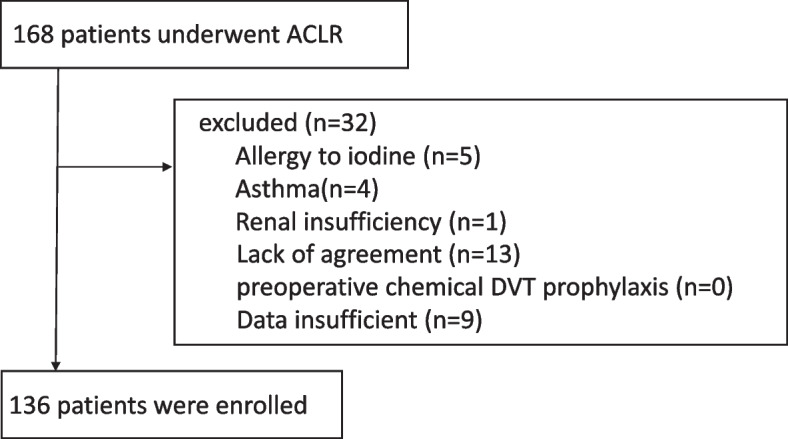
Flowchart of patients included in this retrospective study

Of the 168 patients who were aged > 20 years and who underwent ACL reconstruction, 5, 4, and 1 had an allergy to iodine, asthma, and renal insufficiency, respectively. There was no patient who had given chemical DVT prophylaxis before surgery. Thirteen patients declined to participate in the study and nine did not undergo postoperative D-dimer test. After excluding these patients from this study, the remaining 136 patients (knees) were included in this retrospective comparative study (Fig. 2).

The baseline characteristics of the subjects are summarized in Table 1. Among the 136 patients enrolled in the study, 15 (11.0%) had radiographic VTE; 7 (5.1%) had PE, 5 (3.7%) had DVT, 3 (2.2%) had PE and DVT. Only 1 of the 15 patients showed symptomatic DVT with Homan’s sign (Table 2). No detectible statistical difference between the non-VTE and VTE groups was found for age, sex, BMI, body side, operation technique, autograft, operation time, and tourniquet time. The postoperative D-dimer level was significantly higher in the VTE group than in the non-VTE group.

**Table 1:** Clinical risk factors associated to VTE

Parameter	Total *N* = 136 (SD or %)	VTE (−) *N* = 121 (SD or %)	VTE (+) *N* = 15 (SD or %)	*P*-value
Age (year)	32.5 (10.8)	32.1 (10.9)	35.7 (9.6)	.226
Gender (Male/Female)	88 (64.7) /48 (35.3)	76 (62.8) / 45 (37.2)	12 (80.0) / 3 (20.0)	.257
BMI (kg/m^2^)	24.8 (4.1)	24.8 (4.1)	24.9 (4.4)	.915
Smoke	23 (18.9)	21 (19.4)	2 (14.3)	1.00
Operation technique				.201
ACLR	71 (52.2)	64 (52.9)	7 (46.7)	
ACLR + Meniscal repair	42 (30.9)	36 (29.8)	6 (40.0)	
ACLR + Meniscectomy	12 (8.8)	12 (9.9)	0 (0)	
ACLR+MCLR	3 (2.2)	1 (0.8)	2 (13.3)	
ACLR+ORIF	1 (0.7)	1 (0.8)	0 (0)	
ACLR revision	7 (5.1)	7 (5.8)	0 (0)	
Graft				.444
ST	106 (77.9)	93 (76.9)	13 (86.7)	
BTB	11 (8.1)	10 (8.3)	1 (6.7)	
QT	14 (10.3)	14 (11.6)	0 (0)	
ST + G	5 (3.7)	4 (3.3)	1 (6.7)	
Operation time (min)	192.8 (49.7)	193.6 (50.3)	186.9 (45.4)	.627
Tourniquet time (min)	31.3 (22.8)	31.4 (22.5)	30.9 (26.6)	.947
Preoperative blood test				
WBC (10^2^/μl)	61.7 (13.4)	61.3 (13.6)	65.1 (11.3)	.294
Hb (g/dl)	14.8 (1.6)	14.8 (1.6)	14.8 (1.5)	.980
Plt (10^4^/μl)	25.3 (4.8)	25.3 (4.8)	25.0 (4.9)	.782
PT-INR	0.96 (0.05)	0.96 (0.05)	0.96 (0.06)	.936
D -dimer (μg/ml)	0.59 (0.21)	0.58 (0.22)	0.64 (0.21)	.552
Postoperative blood test				
D -dimer (μg/ml)	2.16 (1.3)	2.01 (1.2)	3.41 (1.0)	<.001

*VTE* Venous thromboembolism, *BMI* Body mass index, *ACLR* Anterior cruciate ligament reconstruction, *MCLR* Medial collateral ligament reconstruction, *ORIF* Open reduction and internal fixation, *ST* Semitendinosus tendon, *BTB* Bone-tendon bone, *QT* Quadriceps tendon, *G* Gracilis, *WBC* White blood cell, *PT-INR* Prothrombin time-international normalized ratio

**Table 2:** Characteristics and Treatment of Patients who had VTE

Age, y	Gender	DVT/PE	Symptom	Medication, period	contrast-enhanced CT	Post D-dimer, μg/ml
39	Male	DVT	asymptomatic	Apixaban, 6 M	Not performed	4.5
47	Female	DVT	asymptomatic	Warfarin, 3 M	Disappear	3.7
24	Male	DVT	asymptomatic	Warfarin, 3 M	Not performed	1.7
39	Male	PE	asymptomatic	Warfarin, 3 M	Disappear	4.8
43	Male	PE	asymptomatic	Warfarin, 1 M	Disappear	4.2
27	Male	PE	asymptomatic	Warfarin, 1 M	Disappear	2.9
31	Male	PE and DVT	asymptomatic	Edoxaban Tosilate Hydrate, 3 M	Disappear	4.4
24	Male	PE	asymptomatic	Apixaban, 3 M	Disappear	4.2
37	Male	PE and DVT	asymptomatic	Edoxaban Tosilate Hydrate, 3 M	Not performed	3.7
50	Female	PE	asymptomatic	Apixaban, 3 M	Not performed	2.8
23	Female	DVT	Homan’s sign+	Rivaroxaban、3 M	Disappear	3.7
47	Female	DVT	asymptomatic	Apixaban, 3 M	Disappear	3.3
23	Female	PE and DVT	asymptomatic	Apixaban, 3 M	Not performed	3
39	Male	DVT	asymptomatic	Apixaban, 3 M	Disappear	1.8
43	Male	DVT	asymptomatic	Apixaban, 3 M	Not performed	2.5

*VTE* Venous thromboembolism, *DVT* Deep vein thrombosis, *PE* Pulmonary embolism

Multivariate logistic analyses showed that the postoperative D-dimer level (OR = 1.85, *P* = .002) significantly increased the risk of VTE (Table 3).

**Table 3:** Multivariate Logistic Regression Analyses of Risk Factor in VTE

	Odds ratio (95% CI)	*P*-value
Age, > 40	1.24 (0.35–4.42)	.739
Gender, female	1.72 (0.41–7.05)	.454
Postoperative D-dimer	1.85 (1.25–2.72)	.002

*VTE* Venous thromboembolism, *CI* Confidence Interval

**Fig. 3:**
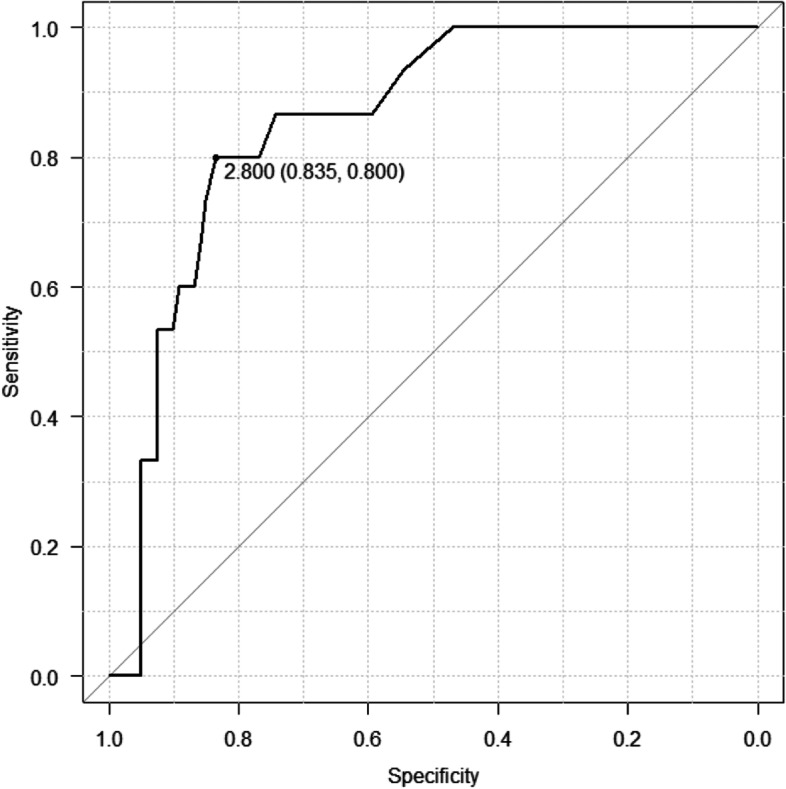
Receiver operator characteristic (ROC) curve of postoperative D-dimer levels as a factor related to postoperative VTEs. The area under the ROC curve was 87.5%. Based on ROC curve analysis, a cutoff D-dimer value of 2.8 mg/mL was deemed best to separate developing VTEs (sensitivity, 80.0%; specificity, 83.5%; *P* = .001)

The sensitivity and specificity of the postoperative D-dimer test were calculated using cutoff values. The ROC curve analysis determined that the AUC of the postoperative D-dimer test was 0.875. A cutoff value of 2.8 mg/mL of the postoperative D-dimer level resulted in the highest sensitivity and specificity, that is, 80.0 and 83.5% (Fig. 3).

## Discussion

The most important findings of this study were that the incidence of radiographical VTE after ACLR with mechanical prophylaxis was 11.0%, as diagnosed by contrast-enhanced CT, and elevated D-dimer level 7 days after surgery, was an independent predictor of VTE in patients undergoing ACLR according to multivariate analysis. The optimal cutoff value for postoperative D-dimer level was 2.8 μg/ml, according to the ROC curve analysis, with a sensitivity of 80.0% and specificity of 83.5%. Information in the present study is clinically important because detection of all VTEs after ACLR is difficult without contrast-enhanced CT.

The ability of contrast-enhanced CT to detect DVT is comparable with that of Doppler sonography in symptomatic patients, which implies that contrast-enhanced CT is an alternative modality, at least in symptomatic patients or patients with a clinical suspicion for a DVT [22–24]. Seung-Ick Cha et al. [21] suggested that the incidence of proximal DVT was 5.5% and the overall incidence of CT-angiographic PE was 6.6% in major orthopedic surgery. Okada et al. [31] performed contrast-enhanced CT after TKA and found asymptomatic VTE in 61.3% of patients without thromboprophylaxis. Gandhi et al. [32] reported that contrast-enhanced CT-detected asymptomatic PE in 41% of the patients after TKA surgery with low-molecular-weight heparin. In this study, the incidence of DVT and PE was 6.2 and 5.4% 7 days after ACL reconstruction with mechanical prophylaxis. Symptomatic PE occurs relatively rarely after ACL reconstruction, with incidence ranging from 0.05 to 0.21% based on a large database analysis [7, 8, 33]. Nagashima et al. [11] reported that at least 7.3% of asymptomatic PEs were detected after ACLR with contrast-enhanced CT, which was suspected to be a higher incidence than that of symptomatic PEs. In this study, the incidence of asymptomatic PEs was 5.4% with contrast-enhanced CT, which was similar to the incidence reported in a previous study [11]. Age, sex, tourniquet time, multiple ligament injury, and immobilization time were reported as risk factors for developing VTE after ACLR. Regarding age, the finding of older age being a risk factor for VTE was previously reported in several series of patients undergoing ACL reconstruction [7, 8, 33, 34]. Kraus et al. [8] reported that the only independent risk factor for symptomatic VTE was age of ≥40 years. In this study, age was not a risk factor of radiographic VTE, including asymptomatic cases after ACL reconstruction in multivariate analysis. There might be several reasons that this study may have been underpowered for an analysis between age and VTE, and this study also included only Japanese patients who generally have a small population of obese patients. The average BMI was 25.0 kg/m^2^ in this study, which was relatively lower than the BMI values reported for individuals of other races.

The hormonal status of female patients or the use of contraceptives could render them susceptible to DVT. Ye et al. [30] reported that female patients have a significantly higher risk of developing DVT after ACLR. They suspected that the female population had a greater mean age (35.8 ± 11.9 years) than their male counterparts (27.8 ± 8.1 years); however, a multivariate analysis was not used in their study. In this study, sex was not a risk factor for VTE after ACL reconstruction according to a multivariate analysis. DVT was more frequent with tourniquet times of > 2 h, and an extended tourniquet time was associated with combined ACL reconstruction and concomitant surgery involving the PCLR [3, 6]. In this study, there was no difference between prolonged tourniquet time and additional surgery in univariate and multivariate analyses, which could be owing to the relatively short average tourniquet time, that is, 32 min during graft harvesting, and the surgical approach being meniscal suture. The majority of the additional surgeries were meniscal repairs, and only 2.5% of this series included multiligament reconstruction. Postoperative bed rest and immobility are the known risk factors for DVT and PE and could be attributed to inpatient surgeries [35]. The ideal duration of knee immobilization after ACL reconstruction has not been elucidated, although the reported duration varied from 0 to 7 days [30, 36, 37]. From the aspect of immobilization, although the present study had no control or alternative condition groups except 1 week immobilization groups, the incidence of radiographic VTE was similar to that reported in previous studies [9, 30, 38].

D-dimer level has been reported to be a negative predictor for DVT. Ota et al. [39] reported the diagnostic value of preoperative D-dimer level to be 1.8 mg/mL (the cutoff value) with a sensitivity of 95% and specificity of 61.9% for orthopedic conditions. However, it has been demonstrated that surgery, trauma, hemorrhagic disease, inflammatory disease, liver and kidney diseases as well as pregnancy, which activates the internal fibrinolytic system, cause elevated levels of plasma D-dimer [40, 41]. Previous reports have demonstrated that D-dimer level was a useful screening test to exclude DVT after orthopedic surgery [42, 43]. Yoo et al. [42] reported the diagnostic value of D-dimer level to be 3.33 mg/mL on postoperative day 7 after THR with a sensitivity of 87% and specificity of 67%. Jiang et al. [43] reported that D-dimer level could predict the development of DVT with the highest sensitivity of 71.4% and specificity of 81.7% at the cutoff value of 6.17 mg/mL on postoperative day 7 for orthopedic surgeries, including TKA, THR, spinal column decompressive internal fixation, and hip fracture reduction internal fixation. In this study, the diagnostic value of D-dimer levels to predict VTE was 2.8 mg/mL on postoperative day 7 after ACL reconstruction with a sensitivity of 92.3% and specificity of 82.2%. Thus, it seemed that D-dimer levels were a useful tool to exclude VTE of patients after ACL reconstruction.

Certain limitations in this study must be considered. First, the data were not compared with conventional pulmonary angiography and venography or sonography of the lower extremities, which are the gold standard for the diagnosis of PE and DVT. Second, this study was a retrospective study, and the number of cases was relatively small after ACL reconstruction; however, the sample size was adequate for the detection of significant difference of postoperative D-dimer between the VTE negative and VTE positive patients. Third, the absence of VTE screening such as contrast-enhanced CT, venography or sonography before surgery is a possible limitation of the present study. The patients who underwent ACL reconstruction in this study rarely had severe complications before surgery; however, the presence of preoperative VTEs was never evaluated.

## Conclusion

The incidence of ACLR-associated radiographical VTEs (DVT and PE) with mechanical prophylaxis was 11.0% in this study. Elevated D-dimer level at 7 days after surgery is an independent predictor of VTEs in patients undergoing ACLR. The postoperative D-dimer level is a more reliable marker for identifying patients who developed VTE after ACL reconstruction.

## Data Availability

Data associated with this study is retained at the Department of Orthopaedic Surgery, Osaka Metropolitan University Graduate School of Medicine. The datasets generated and/or analyzed in this study can are available from the corresponding author on reasonable request. If there are any questions, please contact the corresponding author.

## Abbreviations

VTEs: Venous thromboembolisms
ACLR: Anterior cruciate ligament reconstruction
CT: Computed tomography
DVT: Deep venous thrombosis
PE: Pulmonary embolism
BMI: Body mass index
PT-INR: Prothrombin time-international normalized ratio
ROC: Receiver operating characteristic
AUC: The area under the curve
